# Modeling hologenome imbalances in inflammation and cancer

**DOI:** 10.3389/fcimb.2014.00134

**Published:** 2014-09-24

**Authors:** Yiorgos Apidianakis, Dominique Ferrandon

**Affiliations:** ^1^Department of Biological Sciences, University of CyprusNicosia, Cyprus; ^2^UPR9022 CNRS, University of Strasbourg Institute for Advanced StudyStrasbourg, France

**Keywords:** *Drosophila*, human, mouse, innate immunity, microbiota, hologenome, diet, aging

Genetics play a pivotal role in cancer. This is best exemplified in sporadic intestinal cancer development, which usually starts with mutations in APC then in Ras, p53 and TGFβ (Sears and Garrett, 2014). Nevertheless, intestinal bacteria, diet and lifestyle contribute significantly to mucosal inflammation and cancer (Anand et al., 2008; Kostic et al., 2014; Sears and Garrett, 2014). An effective approach to study the aforementioned factors may be to analyze them combinatorially. In this regard, intestinal dysbiosis is a useful concept to describe harmful changes in the constitution of the microbiota. Another imbalance occurs during inflammatory bowel disease due to an excessive immune response to the intestinal microbiota, which in turn may lead to dysbiosis and perpetuate inflammation. Suspected factors, such as immune system mutations or tissue-damaging microbial strains, may not suffice to promote inflammation and cancer in the absence of co-founding factors that create or sustain an imbalance. Thus, a broad unifying concept may describe disease as dysfunctional interactions among environmental factors, such as diet and lifestyle, microbiota composition, and the genetic of the host. Moreover, aging affects the onset of inflammation and cancer, the host microbiota and the occurrence of sporadic mutations. Accordingly, the host genetic background and that of the microbiome, define the intestinal hologenome, which is influenced by age and the environment toward homeostasis or disease. Thus, intestinal disease may ensue when the intestinal hologenome is imbalanced, that is, when a genetically predisposed or old host interacts with its dysbiotic microbiota in an inadequate or harmful dietary or lifestyle-shaped environment.

The review and opinion articles accompanying this editorial describe key aspects of modeling the hologenome with an emphasis on intestinal infection, inflammation and cancer. One major issue discussed is the adaptation of Koch's postulates in order to assess causation between the human opportunistic pathogen *Pseudomonas aeruginosa* and intestinal disease in patients with cancer (Markou and Apidianakis, 2014). While *Enterobacteriaceae* are suspected contributors to intestinal inflammation and cancer, *P. aeruginosa* exemplifies the opportunistic nature of many bacterial species toward colonization and disease. The suggested guidelines therefore provide a simple framework within which clinical associations, experimental data, and improved outcomes upon treatment against suspected bacteria need to be taken into account in order to prove causation.

Experimental data can be obtained with the various mouse models of intestinal inflammation and cancer described comprehensively by Gkouskou et al. (2014). This review article describes the contributing role of microbiota as a whole, as well as that of specific bacterial species in exacerbating the disease. Interestingly, *Enterobacteriaceae* and *Bacteroides* species contribute to disease progression in various mouse models. In addition, intestinal dysbiosis is influenced by diet, antibiotics, and an immune genetic background conducive to exacerbated adaptive and diminished innate immune response. The authors highlight the potential of targeting the dysbiosis-inflammation-tumorigenesis axis for the development of novel therapeutic strategies for IBD and colorectal cancer.

Whereas studies on bacteria dominate the literature on the role of dysbiosis in inflammation and cancer, viruses were historically the first microbes to be linked to cancer. A modern approach to this issue is described by Iacovides and colleagues who suggest that the interplay between cancer and cell stemness can be influenced by oncogenic viruses (Iacovides et al., 2013). These viruses interfere with signaling pathways that are traditionally associated with self-renewal and lineage-commitment. Thus virus-associated cancers can serve as models to understand the link between viral infection, cancer, and stemness.

Innate immune and stress responses lie at the intersection of apoptosis and cell proliferation during inflammation and cancer. In this regard the simple model organism *Caenorhabditis elegans* has provided valuable insights into the tight regulation of apoptosis during development, infection, and DNA damage (Arvanitis et al., 2013). These findings have been taken a few steps further with the use of *Drosophila* models of infection and cancer, as reviewed by Bangi (2013). This review illustrates the key role of stress, innate immunity, and inflammatory signaling pathways in promoting intestinal stem cell proliferation and tumorigenesis. Prominent among these pathways is the c-Jun-N-terminal kinase (JNK) cascade, which in an oncogenic background can be diverted from tissue damage- or infection-mediated apoptosis to tumor cell proliferation and invasion (Apidianakis et al., 2009; Cordero et al., 2010; Bangi et al., 2012). Ligoxygakis and colleagues contribute a thorough review on *Drosophila* hemocytes, describing the multifaceted roles of these innate immunity cells in development, immunosurveillance, and tumorigenesis (Wang et al., 2014). Kim and Lee explain the multiple roles of *Drosophila Duox*, an NADPH oxidase, the homologs of which mediate bacterial killing via oxygen radicals in mammalian mucosae and phagocytes (Kim and Lee, 2014). The authors provide insights into the role of *Duox* in gut immunity, homeostasis of the intestinal epithelium, and stem cell proliferation. Complementarily, Ayyaz and Jasper put in perspective aging and three responses of *Drosophila* to intestinal microbes, namely, *Duox*, the Immune deficiency NF-κ B pathway, and the renewal of intestinal enterocytes (Ayyaz and Jasper, 2013). These two reviews provide a comprehensive analysis of intestinal dysbiosis and accompanying intestinal cell renewal, which is a homeostatic arm of the intestinal host defense induced either by pathogenic or seemingly innocuous bacteria, and showcase the usefulness of *Drosophila* as a model for the study of intestinal immunity, inflammation, and disease.

Regenerative and tumor-promoting cytokines in *Drosophila* and mammals may not necessarily emanate from tissue infiltrating blood cells (Panayidou and Apidianakis, 2013; Gkouskou et al., 2014). The review by Kux and Pitsouli highlights that regeneration signals are not confined to the *Drosophila* intestinal epithelium (Kux and Pitsouli, 2014). Neighboring tissues, such as muscles, trachea and potentially the neural system communicate with intestinal epithelial cells, and thus might contribute to the intestinal stem cell niche. Accordingly, regenerative or tumor-promoting inflammatory signaling may be controlled not only by tumors and their microenvironment, but also by remote organs. Taking a far-reaching perspective, Droujinine and Perrimon provide an educated guess on the tissues that may systemically provide inflammatory and other inter-organ signals either locally or systemically (Droujinine and Perrimon, 2013). The authors foresee the existence of a vast inter-organ communication network (ICN) of peptides, proteins, and metabolites that act in-between organs to coordinate cellular processes, either under homeostatic or stress conditions. A unique strength of the *Drosophila* model is that biochemical studies can be combined to *in vivo* genome-wide organ-specific genetic screens to identify ICN components.

## Conflict of interest statement

The authors declare that the study was conducted in the absence of any commercial or financial relationships that could be construed as a potential conflict of interest.
